# Effects of Elastic Bands, Kaatsu Cuffs, and Clinical Cuffs on the Brachial Blood Flow during Elbow Flexion Exercise

**DOI:** 10.5114/jhk/193490

**Published:** 2024-12-19

**Authors:** Rodrigo Volga Fernandes, Roque Santos de Oliveira, Luciana Diniz Nagem Janot de Matos, Alexandra Passos Gaspar, Gilberto Laurentino

**Affiliations:** 1 Blood Flow Restriction and Exercise Research Group, São Judas University, São Paulo, Brazil.; 2 Hospital Israelita Albert Einstein, São Paulo, Brazil.

**Keywords:** resistance training, blood flow restriction therapy, regional blood flow, blood flow velocity, ischemia

## Abstract

The elastic band (EB) may be an alternative for restricting the blood flow compared to the Kaatsu and clinical cuffs (KA and CC, respectively). However, the impact of the EB, the KA, and the CC on the blood flow during exercise remains uncertain. This study examined the blood flow (BF), the diameter of the brachial artery (DA), and blood flow velocity (BFV) during elbow flexion exercises using the KA, the CC, and the EB. Twenty-six resistance-trained men (age: 30.7 ± 8.7 years, body mass: 80.7 ± 15.5 kg, body height: 175.7 ± 6.5 cm) completed four sets of 15 repetitions of unilateral elbow flexion exercise at 20% 1RM. All protocols were set to the same perceived tightness (PT) based on Kaatsu optimal pressure (KOP). The BF, BFV and the DA were assessed at baseline, at KOP, and during the exercise sets. The BF and BFV were significantly reduced from baseline to KOP when the KA (67%, ES: 1.4, p = 0.0002; 24%, ES: 1.9, p < 0.0001) and the CC (70%, ES: 1.7, p < 0.0001; 31%, ES: 1.6, p < 0.0001) were applied, yet not the EB (49%, ES: 1.1, p = 0.103; 17%, ES: 0.7, p = 0.123). The BF and BFV increased from KOP to the fourth exercise set in all protocols with the KA (409%, ES: 2.4, p < 0.0001; 37.5 %, ES: 1.7, p = 0.007), the CC (377%, ES: 2.0, p < 0.0001; 55%, ES: 1.3, p < 0.0001) and the EB (411%, ES: 2.9, p < 0.0001; 43%, ES: 1.3, p = 0.002), respectively, with no significant difference between them (p > 0.05). The DA remained unchanged after all protocols (p > 0.05). In conclusion, the EB showed similar blood flow behavior compared to pressure-controlled cuffs.

## Introduction

Low-load (LL) resistance training combined with blood flow restriction (BFR) has been shown to be an effective method to induce increases in strength and muscle hypertrophy compared to high-load resistance exercise (Lixandrão et al., 2018; Xiaolin et al., 2023). The benefits of BFR to skeletal muscular adaptations seem to be a dose-response of restriction pressure. The cuff pressure recommended to induce BFR benefits ranges from 40% to 80% of arterial occlusion pressure (AOP) (Patterson et al., 2019). This level of restriction creates an environment of low oxygen availability and high accumulation of metabolites (Suga et al., 2012), increasing motor unit recruitment, GH concentrations, and intracellular signaling of proteins that regulate protein synthesis and angiogenesis (Bichowska-Paweska et al., 2024; Fujita et al., 2007; Loenneke et al., 2015; Takarada et al., 2000; Volga Fernandes et al., 2022).

In general, different types of cuffs have been used for restricting the blood flow in BFR protocols. For instance, the Kaatsu cuff (KA) is a pneumatic cuff connected to a pressure device, which in combination with either resistance training, ischemic preconditioning (IPC) or aerobic training induces increases in muscle mass, strength, and functionality in both young and elderly individuals (Abe et al., 2010; Slysz et al., 2019; Takarada et al., 2000). On the other hand, the clinical cuff (CC) has been used as an alternative for restricting the blood flow in bedridden individuals (Kubota et al., 2008) and/or during exercise protocols (Laurentino et al., 2016). Prior to starting the BFR protocols, a sphygmomanometer with a nylon cuff is positioned on the lower body with an individualized or an absolute pressure (Ellefsen et al., 2015). However, when the individualized pressure is required from participants, a portable Doppler becomes necessary, rendering the CC technique less accessible to professionals. Consequently, new methods for adjusting pressure or exploring alternative cuff types have been studied (De Oliveira et al., 2024; Duarte Oliveira et al., 2023; Loenneke and Pujol, 2009; Wilson et al., 2013).

In this regard, because of the accessibility and relative cost-effectiveness of KA and CC, the use of elastic bands (EB), also referred in some studies as “practical blood flow restriction” [pBFR] (Lowery et al., 2014; Wilson et al., 2013), has been initially proposed as a viable alternative for restricting the arterial and venous blood flow, with low cost and easy accessibility (Loenneke and Pujol, 2009). The practical application of blood flow restriction entails using an elastic band placed proximally on the arms or legs to restrict the arterial blood flow, in a similar fashion to traditional cuffs. Tightness adjustment methods for this type of the cuff vary, including the deformation level of the elastic material (Behringer et al., 2017), limb circumference (Abe et al., 2019), a tightness perception scale (Wilson et al., 2013), or the rating of perceived pain (Duarte de Oliveira et al., 2023), each offering its own set of advantages and disadvantages.

Some studies have investigated the acute (Loenneke et al., 2010, 2012) and chronic effects of pBFR (Bjornsen et al., 2018; Lowery et al., 2014; Lubbers et al., 2019; Yamanaka et al., 2012). Acutely, most studies have shown that the use of elastic bands induced higher levels of fatigue and subjective perception of effort when compared to the same condition without restriction (Loenneke et al., 2010, 2012). Additionally, other studies have observed significant increases in strength and hypertrophy chronically. For example, in a study conducted by Lubbers et al. (2019), they observed superior increases in squat exercise strength in the group that performed a low-intensity protocol (30% 1RM) with pBFR, compared to a group with the same protocol without restriction. Similar results were found by Yamanaka et al. (2012) in a four-week study on young athletes. Another study conducted on professional powerlifters utilizing two high-frequency blocks in a periodization model of these athletes observed selective hypertrophy in type I fibers and a greater number of myonuclei per fiber in the vastus lateralis muscle only in the group that trained with pBFR (Bjornsen et al., 2018). Considering that the level of blood flow restriction seems to be related to the anabolic response to BFR training (Fry et al., 2010; Fujita et al., 2007), it is important to investigate whether the magnitude of the blood flow restriction among the KA, the CC, and the EB is similar when the cuffs are inflated or tightened at the same perceived tightness.

Thus, this study aimed to examine blood flow variables during and after elbow flexion exercises at 20% 1RM using the Kaatsu cuff, the clinical cuff, and the elastic band adjusted to the same perceived tightness. Our hypothesis posited that the arterial blood flow, blood flow velocity, and the brachial artery diameter would demonstrate similar patterns among the KA, the CC, and the EB both at rest and during the exercise sets.

## Methods

### Participants

Twenty-six recreationally resistance-trained men (age: 30.7 ± 8.7 years, body mass: 80.7 ± 15.5 kg, body height: 175.7 ± 6.5 cm) with at least six-month experience in resistance training were recruited for this study. Participants were excluded from the study if they responded “yes” to one or more questions of the Physical Activity Readiness Questionnaire (PAR-Q), and if their systolic blood pressure was ≥140/90 mmHg; the other exclusion criteria were as follows: smoking, the use of alcohol, thromboembolism history, varicose veins, deep vein thrombosis familiar history, acute myocardial infarction six months prior to the study, cholesterol total ≥220 mg/dl, the use of oral anticoagulants and antiplatelet agents, symptomatic peripheral arterial disease, uncontrolled diabetes, orthopedic and neurologic diseases. All the participants were oriented to maintain their routine of training during the study. Prior to the experiment (~48 h), participants refrained from strenuous exercises for elbow flexor muscles and the use of caffeine, as well as any stimulants for at least 24 h prior to each experimental training session. The sample size estimation indicated the minimum of 33 individuals, assuming statistical power (α = 0.05, and 1-β = 0.95) with an effect size of 0.25 (G* power – 3.1). The study was carried out in accordance with the Declaration of Helsinki, and approved by the Institutional Review Board of the Hospital Israelita Albert Einstein (approval code: SGPP N° 3982-19; approval date: 21 September 2020).

### Experimental Design

We applied a randomized crossover design of the study and participants attended the laboratory five times. During the first and second visits, the procedures of the study were fully explained to participants before they signed informed consent forms. Firstly, the brachial blood flow was assessed before familiarization tests and measurements. Next, participants were familiarized with the one-repetition maximum test (1RM), the Kaatsu cuff (KA), the clinical cuff (CC) and the elastic band (EB), along with the perceived tightness scale (PT) in accordance with Wilson et al. (2013); they were also informed about the procedure to determine arterial occlusion pressure (AOP) using an auscultatory Doppler device, and the exercise protocols.

The brachial blood flow, 1RM and PT measurements from day 1 and day 2 were used for test-retest reliability. From the third to the fifth visit, participants completed one of the three elbow flexion exercise protocols, in random order, separated by at least 72 h. All the protocols were performed in four sets of 15 repetitions at 20% 1RM, with 60-s rest intervals. Given that this study sought to compare the KA with both CC and EB techniques, the perceived tightness at Kaatsu optimal pressure was first measured before each experimental session and then reproduced for performing the KA, CC and EB protocols, in random order, at rest and during the exercise sets on separate days. The blood flow, blood flow velocity and the diameter of the brachial artery were assessed at baseline, at Kaatsu optimal pressure (KOP), after 1, 2, 3 and 4 sets of exercises, and 5 min post-exercise.

### Unilateral Elbow Flexion 1RM Test

The procedures adopted for the unilateral elbow flexion one-repetition maximum (1RM) test followed the recommendations described by Brown and Weir (2001). Briefly, a specific warm-up of the elbow flexor muscles consisted of one set of eight repetitions at 50% of the estimated 1RM, followed by one set of three repetitions at 70% of the estimated 1RM using dumbbells, with a 2-min rest interval between sets. After a 3-min rest period, participants had up to five attempts to achieve their 1RM. A 3-min rest interval was allowed between attempts, and the highest 1RM obtained among attempts was used for further analysis. Typical error (TE), the minimal difference needed to be real (MD), the intraclass correlation coefficient (ICC) and the coefficient of variation (%CV) were 0.4 kg, 1.2 kg, 0.994 and 3.1%, respectively.

### Measure of Perceived Tightness

The measure of perceived tightness (PT) was used to evaluate the blood flow, blood flow velocity peak, and the diameter of the artery before and during the KA, CC, and EB exercise protocols. The perceived tightness scale proposed by Wilson et al. (2013) was initially explained to participants, with “0 out of 10” indicating no tightness, “7 out of 10” indicating moderate tightness with no pain, and “10 out of 10” indicating intense tightness with pain. However, participants could appoint any number on the PT scale. Typical errors (TE) from KA, CC, and EB exercise protocols were 1.6, 1.7, and 1.6 arbitrary units (a.u.), respectively. The minimal differences needed to be real (MD) from KA, CC, and EB protocols were 4.6, 4.6, and 4.4 a.u., respectively. The intraclass correlation coefficients (ICCs) from KA, CC and EB protocols were 0.295, 0.210, and 0.193, respectively. The coefficients of variation (%CV) from KA, CC, and EB protocols were 44.7%, 46%, and 44%, respectively.

### Brachial Blood Flow, Blood Flow Velocity, and the Diameter of Artery Measurements

Blood flow measurements were assessed with the different cuffs following the recommendations described by Thijssen et al. (2011). Brachial artery images were recorded by a two-dimensional ultrasonography device with a spectral Doppler and a linear transducer (L5-12/60, Logic E (R7), GE Medical Systems, China, CO. LTD) with each participant´s right arm supported at the heart level, relaxed, slightly flexed and in abduction, while participants remained in a seated position. Following application of the transmission gel, a wide-band linear array ultrasound probe was used to locate the brachial artery. The Doppler transducer (L5-12/60) in the B pulse mode was placed on the anteromedial aspect of the arm, perpendicular to the axis of the arm, 2–10 cm proximal to the antecubital fold over the artery, and ultrasound variables were set to optimal image acquisition. The angle of insonation was set at ≤60°. For each blood flow measurement, the end-diastolic diameter of the lumen of the brachial artery was measured using a screen caliper, the artery cross-sectional area (CSA) was calculated for each condition, and the blood flow was calculated using manufacturer-provided software (Logic E, [R7]), GE Medical Systems, China, CO. LTD). Blood flow volume (ml/min) data across 15 s and blood flow velocity (cm/s) data from the last ten consecutive cardiac cycles were recorded and averaged for each measurement. All measures were performed offline and by the same researcher in a blinded fashion from protocols. The blood flow (BF) was calculated automatically by the formula BF = average time of velocity × area of the vessel, using manufacturer-provided software on the screen. The typical error (TE), minimal difference needed to be real (MD), the intraclass correlation coefficient (ICC) and the coefficient of variation (%CV) for the brachial blood flow were 32.5 ml/min, 90.1 ml/min, 0.909 and 38.1%, respectively.

### Measurement of the Brachial Arterial Blood Flow with the Kaatsu Technique

After participants had rested for 15 min in a seated position, a Kaatsu (KA) cuff (5-cm wide) was applied at the most proximal portion of the participant´s right arm. The pneumatic cuff was connected to the device (Kaatsu Nano model, Kaatsu Global Inc. Japan). The KA cuff uses standard Kaatsu units (SKU) to measure the pressure applied on the cuff. Weatherholt et al. (2019) used a pressure gauge to establish a conversion from SKU to mmHg, and 1 SKU was found to be equal to 1 mmHg. Initial tightness of the KA air bands was first manually applied on the limbs to determine the base SKU. The base SKU ranged between 20 and 30 SKU for healthy individuals, according to the manufacturer´s instructions. After the base Kaatsu pressure was determined, additional increments of pressure were performed on the Kaatsu device to determine the Kaatsu optimal pressure (KOP). The correct KOP is not so high as to occlude the blood flow, yet high enough so that one achieves a profound “disturbance of homeostasis”. We can confirm the KOP by two steps: 1) colouration and 2) pulsation. Colouration: at KOP, individuals should always have a present capillary refill. The colouration of their skin on their limbs should be pink or beefy red. At KOP, individuals should feel pulsations under the air bands (Sato, 2005). During the base SKU and KOP measurements, participants were asked to rate the perceived tightness according to the perceived tightness scale developed by Wilson et al. (2013). Next, the Kaatsu cuff was deflated, and participants rested for 5 min before starting elbow flexion exercise protocols. Additionally, the perceived tightness obtained during KOP was taken as a reference for blood flow measurements with the clinical cuff and the elastic band. The mean perceived tightness in KA was 4.7 ± 1.9 (a.u.) in accordance with the PT scale, which was equivalent to 138.5 ± 32.7 mmHg, considering that 1 SKU is equal to 1 mmHg (Weatherholt et al., 2019).

### Measurement of the Brachial Arterial Flow with the Clinical Cuff Technique

Before starting the blood flow measurements with the clinical cuff (CC), arterial occlusion pressure (AOP), base SKU and Kaatsu optimal pressure were determined. For AOP measurement, a nylon cuff (6-cm width and 35-cm length) was positioned at the most proximal region of the participant´s dominant arm. Using a portable Doppler device probe (DV-600; Marted) positioned on the radial artery of the arm from participants, the cuff was inflated at the lowest pressure until the auscultatory arterial pulse was interrupted. This point was considered as AOP (172.2 ± 40.3 mmHg), and then, the cuff was deflated. After 10 min, the perceived tightness at Kaatsu optimal pressure was taken from each participant´s arm and matched with the CC. Thereafter, perceived tightness with the CC was started with a pressure of ~40 mmHg, followed by cycles of 5 to 10 mmHg, until participants reported similar perceived tightness recorded at Kaatsu optimal pressure, with a ~5–10-s interval between cycles. After participants had identified their perceived tightness with the CC, the restriction pressure was recorded (102.7 ± 29.6 mmHg), and the cuff was then deflated. The mean perceived tightness with the CC was 4.5 ± 1.9 (a.u) according to the PT scale. After a 5-min rest interval, brachial blood flow measurements were recorded during exercise with the CC.

### Measurement of the Brachial Arterial Flow with the Elastic Band Technique

Before starting the blood flow measurements with the elastic band (EB), perceived tightness at Kaatsu optimal pressure was taken from each participant´s arm and matched with the EB. After a 5-min rest interval, an elastic band (6-cm width and 53-cm length) was positioned at the proximal region of the dominant arm, and the EB was then tightened with increments of ~1 to 2 cm for 5–10 s until participants reported the level of perceived tightness recorded at Kaatsu optimal pressure. After that, the tightness of the EB was released. The mean perceived tightness with the EB in accordance with the PT scale was 4.0 ± 1.6 (a.u.). After a 5-min rest interval, the brachial blood flow was measured during the exercise with the EB.

### Elbow Flexion Exercise Protocols

Participants completed one of the three unilateral elbow flexion exercise protocols in a randomized crossover design, separated by 72 h, as follows: 1) Kaatsu (KA) cuff; 2) clinical cuff (CC), and 3) elastic band (EB). Before starting the exercise protocols, the perceived tightness at Kaatsu optimal pressure was performed and taken as a reference for the blood flow measurements in KA, CC, and EB protocols. All the protocols were performed at 20% 1RM in four sets of 15 repetitions, with a 60-s rest interval between sets. KA and CC cuffs, as well as the EB, remained inflated/tightened throughout the protocols and rest intervals. The protocols were performed with a cadence of 2 s for the concentric phase and 2 s for the eccentric phase. If participants were unable to perform all target repetitions in each set, the set was ended, and a rest interval was allowed. Blood flow measurements (blood flow volume, blood flow velocity, diameter of the artery) were performed before and during the intervals between sets and 5 min post-exercise.

### Statistical Analysis

Data are presented as means ± SDs. The Shapiro-Wilk and Levene’s tests determined the normality and variance equality of the data. A mixed model with repeated measures was performed for each dependent variable, having as fixed factors the experimental protocols (Kaatsu, clinical cuff, and elastic band) and time (baseline, Kaatsu optimal pressure, 1, 2, 3, and 4 sets, 5 min post-exercise), and participants as a random factor. In addition, when the data presented non-normal distribution and/or missing data, we used the mixed model with three different variance/covariance structures (i.e., autoregressive (AR), compound symmetric (CS), and unstructured (UN)), which chose the smallest BIC (Bayesian information criterion) value for analysis. Whenever a significant F value was obtained, the Tukey´s post hoc test was performed for multiple comparison purposes. Additionally, analysis based on effect size (ES, Cohen´s *d*) was performed by calculating the magnitude of the effect, which was considered trivial [0.2–0.3], moderate [0.4–0.7], or large [≥ 0.8]. The level of significance was set at 5%, and all analyses were performed with the statistical software package SAS 9.2 (SAS Institute Inc., Cary, NC, USA).

## Results

An initial analysis revealed no significant difference among protocols at baseline for all dependent variables (*p* > 0.05). The mean perceived tightness at Kaatsu optimal pressure and with the CC was 4.5 ± 1.9 (a.u.) from 0 out of 10, according to the PT scale by Wilson et al. (2013). The restriction pressure recorded in the CC protocol equaled 59.7% of the participant´s arterial occlusion pressure (102.7 ± 29.6 mmHg). None of the participants was detected to present total arterial occlusion during KA, CC, or EB protocols. The 95% confidence intervals (CI95) and *p* values of differences between conditions/cuffs for the blood flow, blood flow velocity and the diameter of the brachial artery are presented in Table 1.

**Table 1:** Confidence intervals and *p* values for differences between protocols for the blood flow, blood flow velocity and the diameter of brachial artery.

Blood flow (ml/min)							
**Protocols**	**Baseline**	**KOP**	**Set 1**	**Set 2**	**Set 3**	**Set 4**	**5 min**
KA vs. CC	−127.5, 97.6 *p* = 1.000	−111.1, 114.5 *p* = 1.000	−55.14, 172.02 *p* = 0.959	−25.5, 200.2 *p* = 0.395	−51.6, 147.2 *p* = 0.931	−80.7, 145.1 *p* = 1.000	−117.7, 109.8 *p* = 1.000
KA vs. EB	−127.2, 98.5 *p* = 1.000	−95.9, 131.2 *p* = 1.000	−121.2, 104.6 *p* = 1.000	−106.0, 119.8 *p* = 1.000	−115.3, 110.4 *p* = 1.000	−108.5, 117.1 *p* = 1.000	−112.2, 112.9 *p* = 1.000
CC vs. EB	−143.6, 84.9 *p* = 1.000	−95.8, 134.5 *p* = 1.000	−65.1, 165.3 *p* = 0.993	−20.3, 208.8 *p* = 0.278	−55.7, 173.4 *p* = 0.959	−151.0, 78.0 *p* = 0.999	−118.7, 111.5 *p* = 1.000
**Blood flow velocity (cm)s)**							
**Protocols**	**Baseline**	**KOP**	**Set 1**	**Set 2**	**Set 3**	**Set 4**	**5 min**
KA vs. CC	−17.3, 19.5 *p* = 1.000	−12.0, 25.0 *p* = 0.999	−12.4, 24.5 *p* = 0.999	−10.9, 26.0 *p* = 0.997	−10.6, 26.8 *p* = 0.994	−19.1, 18.3 *p* = 1.000	−16.9, 20.4 *p* = 1.000
KA vs. EB	−25.9, 11.0 *p* = 0.997	−19.2, 18.2 *p* = 1.000	−18.7, 18.3 *p* = 1.000	−19.1, 17.9 *p* = 1.000	−19.6, 17.8 *p* = 1.000	−17.1, 20.1 *p* = 1.000	−18.1, 18.7 p = 1.000
CC vs. EB	−25.1, 12.3 *p* = 0.999	−13.0, 24.9 *p* = 0.999	−12.9, 24.6 *p* = 0.999	−11.8, 25.7 *p* = 0.999	−11.6, 25.9 *p* = 0.998	−17.7, 20.0 *p* = 1.000	−16.8, 20.9 *p* = 1.000
**Diameter of artery (cm)**							
**Protocols**	**Baseline**	**KOP**	**Set 1**	**Set 2**	**Set 3**	**Set 4**	**5 min**
KA vs. CC	−0.05, 0.06 *p* = 1.000	−0.05, 0.06 *p* = 1.000	−0.04, 0.07 *p* = 1.000	−0.04, 0.07 *p* = 1.000	−0.04, 0.06 *p* = 1.000	−0.05, 0.06 *p* = 1.000	−0.05, 0.06 *p* = 1.000
KA vs. EB	−0.06, 0.05 *p* = 1.000	−0.03, 0.08 *p* = 0.995	−0.05, 0.06 *p* = 1.000	−0.04, 0.07 *p* = 1.000	−0.04, 0.07 *p* = 0.999	−0.06, 0.05 *p* = 1.000	−0.05, 0.06 *p* = 1.000
CC vs. EB	−0.06, 0.06 *p* = 1.000	−0.03, 0.09 *p* = 0.997	−0.05, 0.07 *p* = 1.000	−0.03, 0.09 *p* = 0.998	−0.03, 0.09 *p* = 0.992	−0.06, 0.06 *p* = 1.000	−0.05, 0.07 *p* = 1.000

Values are expressed as a 95% confidence interval and p value. KA: Kaatsu cuff; CC: clinical cuff; EB: elastic band; KOP: Kaatsu optimal pressure (p < 0.05)

### Blood Flow

**Figure 1:**
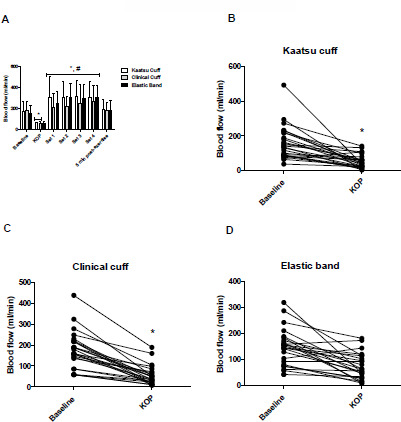
A: Blood flow values (ml/min) [mean ± SD], with the Kaatsu cuff, the clinical cuff and the elastic band at baseline, Kaatsu optimal pressure, sets 1 to 4, and 5 min post-exercise. B, C and D: Individual values of the blood flow (ml/min) from baseline to KOP. * indicates a significant difference from baseline (p < 0.05) # *indicates a significant difference from KOP (p < 0.05) KOP: Kaatsu optimal pressure*

**Figure 2:**
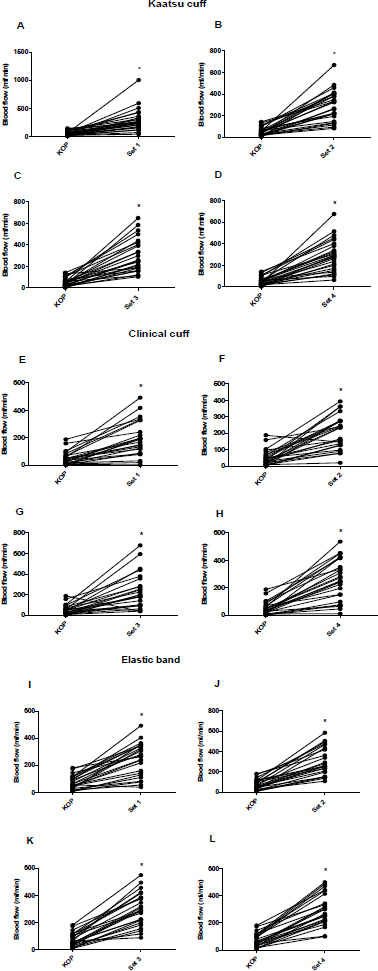
A to L: Individual values of the blood flow (ml/min) for the Kaatsu cuff, the clinical cuff, and the elastic band at sets 1, 2, 3, and 4. * indicates a significant difference from KOP (p < 0.05) KOP: Kaatsu optimal pressure

The brachial blood flow results at different time points are presented in Figures 1 and 2. There was a significant time effect (*p* < 0.0001), without a condition*time interaction (*p* = 0.198) for the blood flow.

The blood flow was significantly reduced from baseline to Kaatsu optimal pressure (KOP) in KA (CI: 29.1 to 183.4 ml/min, −67%, ES: −1.4, *p* = 0.0002) and CC (CI: 44.3 to 201.5 ml/min, 70%, ES: −1.7, *p* < 0.0001) protocols, yet not in the EB protocol (CI: −5.3 to 153.9 ml/min, −49%, ES: −1.1, *p* = 0.103) (Figures 1A–1D). On the other hand, the blood flow was significantly increased in all protocols from KOP to the fourth exercise set (KA: CI: 129.7 to 345.8 ml/min, 409%, ES: 2.4, *p* < 0.0001; CC: CI: 97.2 to 317.4 ml/min, 377%, ES: 2.0, *p* < 0.0001; and EB: CI: 113.6 to 335.2 ml/min, 411%, ES: 2.9, *p* < 0.0001), and during each exercise set in the KA (Figures 2A–2D), the CC (Figures 2E–2H) and the EB (Figures 2I–2L) protocol. In addition, when comparing the baseline to the fourth exercise set, the blood flow was significantly increased in the KA (CI: 21.3 to 241.6 ml/min, 83.4%, ES: 1.0, *p* = 0.003) and the EB (CI: 37.9 to 262.4 ml/min, 105.2%, ES: 1.6, *p* = 0.0004) protocol, but not in the CC (CI: 27.6 to 196.3 ml/min, 47%, ES: 0.68, *p* = 0.451) protocol. There were no substantial differences between cuffs and the EB at Kaatsu optimal pressure or during the exercise protocols for the blood flow (*p* > 0.05) (Table 1). The blood flow returned to the baseline level 5 min after the fourth exercise set in all the protocols (*p* < 0.0001). The typical error (TE), minimal difference needed to be real (MD), and the coefficient of variation (%) of the brachial blood flow were 32.5 ml/min, 90.1 ml/min, and 6.3%, respectively.

### Blood Flow Velocity

**Figure 3:**
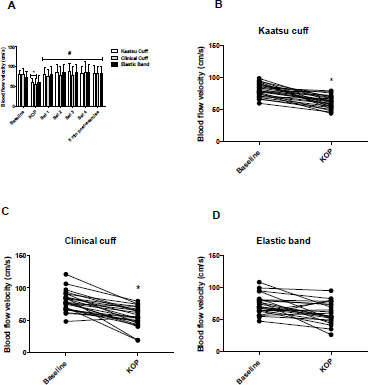
A: Blood flow velocity values (cm/s) [mean ± SD], with the Kaatsu cuff, the clinical cuff and the elastic band at baseline, KOP, sets 1 to 4, and 5 min post-exercise. B, C, and D: Individual values of blood flow velocity (cm/s) from baseline to KOP. * indicates a significant difference from baseline (p < 0.05) ^#^ indicates a significant difference from KOP (p < 0.05) KOP: Kaatsu optimal pressure

The blood flow velocity results at different time points are presented in Figure 3A. There was a significant time effect (*p* < 0.0001), without a condition*time interaction (*p* = 0.640) for blood flow velocity. Blood flow velocity was significantly reduced from baseline to Kaatsu optimal pressure (KOP) in KA (CI: 6.3 to 31.5 cm/s, 24%, ES: 1.9, *p* < 0.0001) and CC (CI: 11.5 to 37.2 cm/s, 31%, ES: 1.6, *p* < 0.0001) protocols, while not in the EB protocol (CI: 1.11 to 25.2 cm/s, 17%, ES: 0.7, *p* = 0.123) (Figures 3A–3D). On the other hand, blood flow velocity was significantly increased in all protocols from KOP to the fourth exercise set (KA: CI: 5.4 to 41.0 cm/s, 37.5%, ES: 1.7, *p* = 0.007; CC: CI: 12.0 to 48.3 cm/s, 55%, ES: 1.3, *p* < 0.0001; and EB: CI: 7.0 to 43.5 cm/s, 43%, ES: 1.3, *p* = 0.002). There were no substantial differences between cuffs and the EB at Kaatsu optimal pressure or during the exercise protocols for blood flow velocity (*p* > 0.05). Blood flow velocity returned to the baseline level 5 min after the fourth exercise set in all the protocols (*p* < 0.0001). The typical error (TE), minimal difference needed to be real (MD), and the coefficient of variation (%) of the blood flow velocity were 11.5 cm/s, 31.7 cm/s, and 2.3%, respectively.

### Diameter of the Brachial Artery

**Figure 4:**
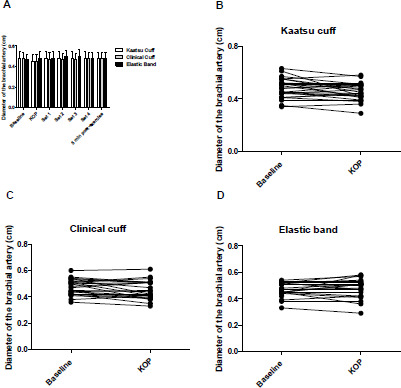
A: Diameter of the brachial artery values (cm) [mean ± SD], with the Kaatsu cuff, the clinical cuff and the elastic band at baseline, KOP, sets 1 to 4, and 5 min post-exercise. B, C and D: Individual values of the diameter of the brachial artery (cm) from baseline to KOP (*p* < 0.05). *KOP: Kaatsu optimal pressure*

The diameter of the artery results at different time points are presented in Figure 4A. The diameter of the brachial artery remained unchanged at Kaatsu optimal pressure and during the exercise sets in all protocols (*p* > 0.05). The typical error (TE), minimal difference needed to be real (MD), and the coefficient of variation (%) of the diameter of the brachial artery were 0.04 cm, 0.1 cm, and 0.5%, respectively.

## Discussion

The purpose of this study was to investigate variables related to the blood flow during and after unilateral elbow flexion exercise at 20% 1RM, using the Kaatsu cuff, the clinical cuff, and the elastic band with the same perception of tightness. Confirming our initial hypothesis, the blood flow, blood flow velocity, and the brachial artery diameter followed a similar response pattern among blood flow restriction techniques at rest and between exercise sets.

In the present study, we sought to standardize the occlusive stimulus, taking Kaatsu optimal pressure (KOP) as a reference, using a perceived tightness scale developed by Wilson et al. (2013). Our findings showed that the blood flow was significantly reduced when the cuffs were inflated from baseline to KOP, with a large effect size for Kaatsu and clinical cuffs (ES: 1.4 and ES: 1.7, respectively), before starting the exercise protocols. In addition, regarding the EB, although a reduction in the blood flow did not achieve statistical significance (*p* = 0.103), there was a reduction of 49%, with a large effect size (ES: 1.1), and a 2.5-fold higher reduction in the blood flow compared to the baseline value. The level of reduction in the blood flow observed for the EB in the present study was similar to that reached by Mouser et al. (2017) (~46% to 53%) when participants performed four sets of elbow flexion exercise at 30% 1RM combined with 40% to 80% of AOP, using a 5-cm non-elastic nylon cuff. Additionally, the blood flow was reduced in most of participants at KOP, except for one participant in the CC protocol (Figure 1C) and two participants in the EB protocol (Figure 1D). Interestingly, there were no substantial differences between KA and CC protocols compared with the EB protocol. Thus, it is plausible to suggest that applying the EB at the same perceived tightness as for Kaatsu and clinical cuffs would result in a similar occlusive stimulus to the exercised muscle.

To our knowledge, only two studies have investigated the effects of the use of the perceived tightness scale and the EB on arterial occlusion pressure (AOP) and the reduction in blood flow restriction (Abe et al., 2019; Bell et al., 2018). In the first study, Bell et al. (2018), using a large sample size (58 men and 62 women), reported that the mean percentage of arterial occlusion pressure achieved was 86.9% when participants rated 7 out of 10 on a perceived tightness scale for the upper body, wearing a nylon cuff (5-cm wide for the arm). The results from the Bell´s et al. (2018) study regarding AOP were higher than those of our study (86.9% vs. 59.7% of AOP), probably due to the higher level of perceived tightness required on the scale (7 vs. 5 out of 10). In the second study, Abe et al. (2019) reported that the reduction in the brachial blood flow at rest was similar when an elastic cuff was pulled to 10%/20% of its initial length compared to a pressurized nylon cuff (CC) inflated to 40%/80% of AOP. However, the brachial artery blood flow was decreased in a dose-pressure-dependent manner in both the CC (80%>40%) and practical elastic cuffs (20% > 10%). Taken together, the results from previous studies highlight that the blood flow may be directly affected by a higher perceived tightness rate applied on the CC and the EB, which, in part, could explain the similarity of the response of reduction in the blood flow in the KA, CC, and EB protocols at the same perceived tightness (i.e., 5 out of 10) presented in this study.

Regarding blood flow velocity, we observed a significant reduction in the brachial blood flow velocity from baseline to Kaatsu optimal pressure (KOP) in both Kaatsu and clinical cuffs (24% and 31%, respectively), with no significant change in the EB (17%). Contrary to our results, Abe et al. (2019) reported a significant reduction of 2% and 21.5% in peak blood velocity when the EB was pulled to 10% and 20% of its initial length, respectively. The divergence from our results may be related to the use of different methodologies for setting perceived tightness (i.e., perceived tightness scale vs. pulled to 10% to 20% from elastic cuff initial length), beyond the cuff model. It is important to underscore that even though the resting blood flow velocity in the EB protocol at perceived tightness (~5 out of 10) was not significantly reduced in the current study, the percentage of reduction of blood flow velocity (17%) was higher than the coefficient of variation between the two measures from day 1 and day 2 (2.3%). Furthermore, only in four out of 26 participants a reduction in blood flow velocity was not achieved (Figure 3D).

Elbow flexion exercise induced increases in the brachial blood flow immediately after the cuff inflation until the end of the fourth exercise set, with large effect sizes for KA (ES: 2.4), CC (ES: 2.0) and EB (ES: 2.9) protocols. However, increases in the blood flow did not differ among protocols. A plausible explanation for a similar response pattern among protocols during exercise sets may be due to the amount of reduction in the blood flow before the commencement of exercise protocols (KA: 67%, CC: 70%, EB: 49%). Our results may be comparable to those observed by Mouser et al. (2017) who reported that the brachial blood flow was reduced immediately after the CC had been inflated at 40% and 80% of AOP (46% and 53%, respectively), before starting the exercise sets, accompanied by increases in the blood flow up to the fourth exercise set, with no significant difference between pressures. It seems that only a moderate relative pressure is sufficient to induce increases in the blood flow during exercise sets following cuff inflation (Mouser et al., 2017). In the present study, although mechanical compression imposed by the KA cuff, the CC, and the EB may have limited the maximum amount of the blood flow to the upper arm during exercise sets, the blood flow remained elevated compared to baseline and Kaatsu optimal pressure. We suggest that increases in the blood flow during exercise in KA, CC, and EB protocols may have been provoked by increased cardiac output due to an increase in the heart rate rather than stroke volume because of the inhibition of venous return induced by cuffs (Takano et al., 2005).

Finally, we did not observe significant differences in the brachial artery diameter when applying cuff inflation or tightness at rest or during exercise sets. According to our findings, mean perceived tightness (i.e., 5 out of 10) applied in KA, CC, and EB protocols was not sufficient to cause substantial changes in the diameter of the brachial artery. Particularly in the CC protocol, perceived tightness resulted in ~59.7% of AOP. Our results are in line with those of Mouser et al. (2017) and Abe et al. (2019), who also used CCs and EBs during exercise execution. In addition, we may speculate that the measurement of the diameter of the brachial artery, which was made distal to the cuff (~5 cm), near the antecubital fossa, could limit the detection of significant changes in the caliber of the artery.

Although there has been criticism of the use of the perceived tightness scale because it does not provide reliable estimates of a relative individual’s arterial occlusion pressure across days (%CV ~12%) (Bell et al., 2019), our findings provide important reliability for the use of the EB and the perceived tightness scale, with %CVs of 3.4%, 0.1%, and 0.8% for the Kaatsu cuff, the clinical cuff, and the elastic band, respectively. Altogether, our findings suggest that the elastic band could be used as an attractive alternative to Kaatsu and clinical cuffs in blood flow restriction protocols. The use of the elastic band with perceived tightness ≤7 may be useful, cost effective, feasible and an alternative strategy when the restriction pressure is unknown.

## Limitations and Future Perspectives

Certainly, this study is not without limitations. First, the perceived tightness scale was designed for implementation with practical blood flow restriction in lower body exercise (Wilson et al., 2013), and in the present study, we investigated exercise sets for the upper arm. However, it is important to highlight that an individual´s perceived tightness scale could be used to apply a subocclusive pressure without exercise (Bell et al., 2018, 2019). Second, the perceived tightness scale was originally implemented using an elastic band, and we applied the scale with a Kaatsu pneumatic cuff and manually controlled pressure cuff (CC). Although we recognize that the resting arterial occlusion pressure is different between types of cuffs (i.e., nylon vs. elastic) (Buckner et al., 2017), and the cuffs of different materials may slightly alter the perceptual response.

Further investigation is necessary to allow extrapolation and generalization from our conclusions. Nevertheless, we expect that our findings may encourage researchers to investigate exercise-induced muscular adaptations such as strength and skeletal muscle hypertrophy in response to these training methods.

## Conclusions

In conclusion, the present study indicates that the Kaatsu cuff, the clinical cuff, and the elastic band at the same perceived tightness when associated with low-load resistance training induced similar responses in blood flow-related variables at rest and during exercise.
